# Histone deacetylase inhibition reduces cardiac connexin43 expression and gap junction communication

**DOI:** 10.3389/fphar.2013.00044

**Published:** 2013-04-15

**Authors:** Qin Xu, Xianming Lin, Laura Andrews, Dakshesh Patel, Paul D. Lampe, Richard D. Veenstra

**Affiliations:** ^1^Department of Pharmacology, State University of New York Upstate Medical UniversitySyracuse, NY, USA; ^2^Leon H. Charney Division of Cardiology, New York University School of MedicineNew York, NY, USA; ^3^Fred Hutchinson Cancer Research CenterSeattle, WA, USA; ^4^Department of Cell and Developmental Biology, State University of New York Upstate Medical UniversitySyracuse, NY, USA

**Keywords:** gap junctions, connexin43, phosphorylation, connexin40, trichostatin A, vorinostat

## Abstract

Histone deacetylase inhibitors (HDACIs) are being investigated as novel therapies for cancer, inflammation, neurodegeneration, and heart failure. The effects of HDACIs on the functional expression of cardiac gap junctions (GJs) are essentially unknown. The purpose of this study was to determine the effects of trichostatin A (TSA) and vorinostat (VOR) on functional GJ expression in ventricular cardiomyocytes. The effects of HDAC inhibition on connexin43 (Cx43) expression and functional GJ assembly were examined in primary cultured neonatal mouse ventricular myocytes. TSA and VOR reduced Cx43 mRNA, protein expression, and immunolocalized Cx43 GJ plaque area within ventricular myocyte monolayer cultures in a dose-dependent manner. Chromatin immunoprecipitation experiments revealed altered protein interactions with the Cx43 promoter. VOR also altered the phosphorylation state of several key regulatory Cx43 phospho-serine sites. Patch clamp analysis revealed reduced electrical coupling between isolated ventricular myocyte pairs, altered transjunctional voltage-dependent inactivation kinetics, and steady state junctional conductance inactivation and recovery relationships. Single GJ channel conductance was reduced to 54 pS only by maximum inhibitory doses of TSA (≥ 100 nM). These two hydroxamate pan-HDACIs exert multiple levels of regulation on ventricular GJ communication by altering Cx43 expression, GJ area, post-translational modifications (e.g., phosphorylation, acetylation), gating, and channel conductance. Although a 50% downregulation of Cx43 GJ communication alone may not be sufficient to slow ventricular conduction or induce arrhythmias, the development of class-selective HDACIs may help avoid the potential negative cardiovascular effects of pan-HDACI.

## INTRODUCTION

Histone acetyltransferases (HATs) and deacetylases (HDACs) regulate the nuclear protein acetylation–deacetylation cycle that modulates gene expression by altering chromatin condensation and transcription factor [e.g., MEF2 (myocyte enhancer factor-2), p53] activities (Kouzarides, 2000; Yang and Grégoire, 2007; Yang and Seto, 2008; Haberland et al., 2009). Several cytosolic proteins have been identified as substrates for the HAT/HDAC protein acetylation cycle (e.g., tubulin; Piperno et al., 1987; Yang and Grégoire, 2007). There are 11 highly homologous mammalian HDACs containing Zn^2^^+^-dependent catalytic deacetylase (DAC) domains, subdivided into three classes based on their divergent amino- and carboxyl-terminal domains. The class I HDACs (HDAC1–3 and 8) consist primarily of a single DAC with short amino and carboxyl-termini, as does the class IV HDAC (HDAC11; Yang and Seto, 2008). The class IIa HDACs (HDAC4, 5, 7, and 9) possess amino-terminal MEF2 and 14-3-3 protein binding motifs and shuttle between the nucleus and cytosol in a phosphorylation-dependent manner (McKinsey et al., 2000; Zhou et al., 2000; Little et al., 2007). Phosphorylation of HDAC4, 5, and 9 promotes the expression of cardiac prohypertrophic genes and their deletion increases susceptibility to stress-induced hypertrophy (Little et al., 2007; Yang and Seto, 2008; Haberland et al., 2009). HDAC4 is implicated in the regulation of cardiac contractility via muscle LIM protein (MLP) acetylation (Gupta et al., 2008). The class IIb HDAC6 is unique in that it possesses two DAC domains and deacetylates tubulin along with sirtuins (SIRT2) (SIRT1–7 are the NAD-dependent class III HDACs; Hubbert et al., 2002; Haberland et al., 2009). The function of the class IIb HDAC10 is poorly understood.

Histone DAC inhibitors (HDACIs) are known to induce diverse biological responses including apoptosis and cell-cycle arrest (Rasheed et al., 2007). HDACI chemical subgroups include, in order of potency, hydroxamic acids, cyclic peptides, benzamides, electrophilic ketones, and short chain fatty acids. Trichostatin A (TSA) is a biological hydroxamate compound with antibacterial, antifungal, and antiproliferative activities attributed to its HDAC inhibitory activity (Yoshida et al., 1990). Two HDACIs have received FDA approval for treating cutaneous T cell lymphoma (CTCL) and there are>140 ongoing HDACI clinical trials as novel therapeutics for numerous diseases including other forms of cancer, neurodegeneration, inflammation, and heart failure (www.clinicaltrials.gov; Marks and Breslow, 2007; Rasheed et al., 2007; Kazantsev and Thompson, 2008; Haberland et al., 2009; Ryan, 2009; Shakespear et al., 2011; McKinsey, 2012). Vorinostat [VOR, suberoylanilide hydroxamic acid (SAHA), Zolinza^TM^] and romidepsin (depsipeptide, FK-228, Istodax^TM^) are relatively non-selective pan-HDACIs despite possessing differential inhibitory potencies for class IIa HDACs (Rasheed et al., 2007; Bradner et al., 2010). Their broad spectrum HDACI profiles produce dose limiting toxicities such as fatigue, thrombocytopenia, gastrointestinal toxicity, and QT interval prolongation (Rasheed et al., 2007; McKinsey, 2011). Dose-dependent toxicities and increased susceptibility to ventricular arrhythmias associated with some HDACI therapies have emphasized the need to develop isoform selective HDACIs (Shah et al., 2006; McKinsey, 2011).

Vorinostat reduced the occurrence of restraint-induced ventricular arrhythmias in Duchenne muscular dystrophic (mdx) mice (Colussi et al., 2010). However, mdx mouse hearts exhibit reduced cardiac sodium channel (Na_V_1.5) expression and connexin43 (Cx43) lateralization owing to a hyperacetylated condition that was reversed by VOR (Colussi et al., 2010, 2011). In wild-type (wt) mouse hearts, VOR increased Cx43 acetylation and lateralization, consistent with observations from the mdx mouse that increased protein acetylation leads to Cx43 dissociation from N-cadherin (Ncad), zonula occludens-1 (ZO-1), and cardiac intercalated disks, and increased c-Src-dependent Y265 phosphorylation, molecular events known to downregulate Cx43-mediated gap junction (GJ) communication (Colussi et al., 2011). Functional assessment of ventricular electrical coupling is difficult to perform in intact heart, but is directly quantifiable by dual whole cell patch clamp analysis in isolated cardiomyocyte cell pairs (Lin et al., 2005, 2008a, 2010).

This study was performed to examine the functional effects of TSA and VOR on ventricular Cx43 GJs. Previous preliminary findings suggested that Cx43 is acetylated and that treatment of primary neonatal mouse ventricular myocyte cultures with TSA reduced electrical coupling by nearly 50% (Hertzberg and Spektor, 2004; Lin et al., 2008b). We assessed the ability of TSA and VOR to inhibit total ventricular HDAC activity using a fluorimetric assay; measured Cx43 and Ncad mRNA and protein levels; determined the phospho-serine (pSer) state of Cx43; quantified the Cx43 GJ plaque area; and measured the GJ conductance (*g*_j_), transjunctional voltage (*V*_j_) gating, and single ventricular GJ channel conductance (γ_j_) properties in ventricular cardiomyocyte cultures by patch clamp techniques. These results provide the first direct evidence for the functional downregulation of Cx43 GJ-mediated electrical coupling between normal ventricular myocytes by pan-HDAC inhibition. These effects presumably result from indirect and direct effects of HDACI-induced increased protein acetylation on Cx43 expression, GJ assembly, and function.

## MATERIALS AND METHODS

### CELL CULTURE

Newborn C57Bl/6 mice were anesthetized with isoflurane and the hearts excised in accordance with procedures approved by the institution’s Committee for the Humane Use of Animals. Neonatal murine atrial and ventricular tissues were dissociated separately in a Ca^2^^+^- and Mg^2^^+^-free collagenase balanced salt solution (dulbecco’s modified saline [DMS_8_], in mM: NaCl, 116; KCl, 5.4, NaH_2_PO_4_, 1.0; and dextrose, 5.5) containing ≈1 mg/ml of purified collagenase (type II), 5.5 μg/ml deoxyribonuclease I (Worthington Biochemical Corp., Lakewood, NJ, USA), and 1 mg/ml bovine serum albumin (BSA, fraction V, Sigma Chemical Corp., St. Louis, MO, USA; Lin et al., 2005, 2008a, 2010). A total of five 10-min dissociation cycles at 37°C were performed using 5 ml of dissociation solution/cycle. The supernatant from the first cycle was discarded and the supernatant from the remaining four cycles were filtered through a 70-μ cell strainer (Falcon, Franklin Lakes, NJ, USA) into 5 ml of M199 cell culture media supplemented with 10% fetal bovine serum (FBS; Atlanta Biologicals) and 100 U/ml penicillin/streptomycin (Invitrogen). Cell pellets were produced by low speed centrifugation (500 rpm, 5 min) and resuspended in M199/FBS media. The primary cell cultures were enriched for cardiomyocytes by a 30-min differential cell adhesion step. The cell media was collected, pelleted by low speed centrifugation, and resuspended in approximately 1 ml of media per dissociated heart ventricles. Approximately 0.1 ml of the ventricular myocyte cell suspension was added to each 35 mm diameter culture dish for patch clamp electrophysiology experiments. The remainder of the ventricular cell suspension was divided between two 35-mm culture dishes or four 12-wells containing fibronectin-coated coverslips for real-time (RT)-PCR, immunoblotting, immunoprecipitation, or immunostaining procedures. Exact cell counts were obtained with a hemocytometer for plating cells in 96-well plates for fluorimetric HDAC activity assays. The media was exchange daily and 200 μM bromodeoxyuridine (BrDU) was added on culture day 2 to the high density myocyte cultures to inhibit fibroblast proliferation. Stable transfectants of HeLa cells with rat Cx43 (HeLa-Cx43 cells) were prepared and cultured as described for mouse Neuro2a (N2a) cells (Lin et al., 2003).

### HDAC ACTIVITY ASSAYS

**FIGURE 1:**
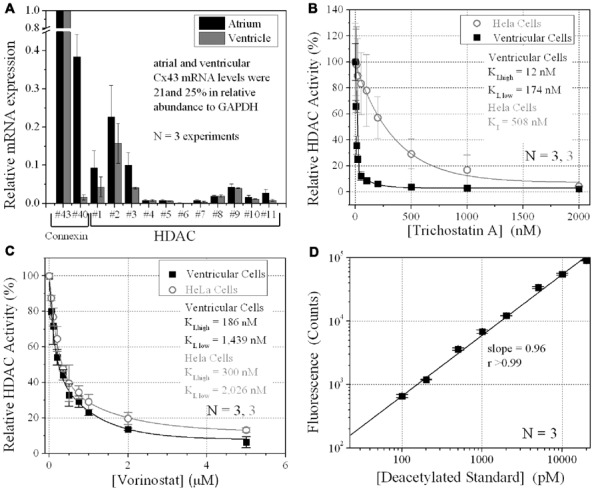
**Ventricular HDAC expression and inhibition**. **(A)** The mRNA expression levels for all 11 mammalian HDACs and two connexins, Cx43 and Cx40, were detected by real-time PCR using the SYBR^®^ GreenER^TM^ qPCR SuperMix (Invitrogen) and custom-designed forward and reverse primers. The data was averaged from three experiments. **(B,C)** Total HDAC activity was measured in ventricular myocyte and stable Cx43-transfected HeLa cell (HeLa-Cx43) cultures by deacetylated Fluor-de-Lys^®^ fluorescence during trichostatin A (TSA) **(B)** or vorinostat (VOR) inhibition **(C)**. All data were normalized to the background subtracted maximum relative fluorescence of the control well. The data from three experiments were averaged and fitted with a second-order exponential decaying function in Origin7.5 and the equilibrium inhibition constants (*K*_I_) were calculated from the expression *K*_I_ = 0.693/[HDACI]decay constant. **(D)** A series of 1:10 dilutions of the deacetylated Fluor-de-Lys substrate were combined with the developer and fluorescence counts were obtained with the BIO-TEK Synergy plate reader in conjunction (i.e., parallel) with the cell-based HDAC activity assays. The background (empty well) subtracted fluorescence emission at 460 nm increased linearly with the concentration of the deacetylated substrate. The experiments were performed in triplicate (mean ± SEM).

Aliquots of 6 × 10^+^^5^ ventricular myocytes or HeLa cells per well (96-well plate) were grown in 200 μl of 200 μM BrDU/M199 or N2a culture media, exchanged daily. Cells densities were counted with a hemocytometer. Cell wells were incubated with 2,000 pmol of the acetylated Fluor-de-Lys^®^ substrate for 6 h during HDAC inhibition. Cell, media, and standard curve deacetylated substrate sample wells were developed according to manufacturer’s directions and background subtracted relative fluorescence unit (RFU) counts were acquired with a BIO-TEK Synergy plate reader (360 nm excitation, 460 nm emission). A standard curve was generated using serial 1:10 dilutions of the deacetylated Fluor-de-Lys standard and developer supplied with the BML-AK503 HDAC fluorometric cellular activity assay kit (**Figure 1D**; Enzo Life Sciences). TSA was purchased from Calbiochem or Enzo Life Sciences. VOR (SAHA, Zolinza^TM^) was initially obtained from Merck HDAC Research, LLC via a Material Transfer Agreement (MTA) for *in vitro* use only and was subsequently purchased commercially from Selleck Chemicals, LLC.

### REAL-TIME PCR

Total atrial or ventricular RNA was isolated with Qiagen RNeasy^®^ mini kit, quantified by UV absorption, and 500 ng reverse-transcribed with QuantiTect^®^ Reverse Transcription kit (Qiagen). Fifty nanograms of the cDNA reaction mix was combined with equal (nM) amounts of custom forward (5′–3′) and reverse (3′–5′) RT-PCR primers, Superscript^®^ enzyme mix, and SYBR^®^ GreenER^TM^ dye in a 200-μl PCR tube (rxn volume = 25 μl). All RT-PCR reagents were from Invitrogen and the samples were run for 40 cycles in a 96-well plate. All results were expressed relative to glyceraldehyde 3-phosphate dehydrogenase (GAPDH) and a cellular RNA sample without reverse transcription was run as a negative control to test for genomic DNA. cT values were determined by the apparatus and the quality of the PCR product was confirmed by analyzing the melt-curve. RT-PCR primers were custom-designed for all murine11 HDAC genes based on the mouse gene sequences according to published rat HDAC RT primers (Morrison et al., 2006), *Gja1*, *Gja5*, *Gapdh*, and *Cdh2* (Ncad) murine genes (Lin et al., 2010). RT-PCR primers were designed to span exon–intron regions of the gene of interest or by custom ordering primer sets from realtimeprimers.com.

Oligonucleotide primer sequences for the RT-PCR assays of murine HDAC1-11, Cx43, Cx40, Ncad, and GAPDH gene expression were: *Hdac1*, forward 5′-TGGGGCTGGCAAAGG CAA-3′, reverse 5′-TGGGGCAGCATCCTCAAGTCC-3′; *Hdac2*, forward 5′-CGGACAAAAG AATTTCCATTCG-3′, reverse 5′-CAATGTCCTCAAACAGGGAAG-3′; *Hdac3*, forward 5′-CCGCTTCCATTCTGAGGACTAC-3′, reverse 5′-GACCCGGTCAGTGAGGTAGAAG-3′; *Hdac4*, forward 5′-GTTCCAGCGTCAACATGAG-3′, reverse 5′-GTTGAGAACAAACTCCTG CAGCT-3′; *Hdac5*, forward 5′-GCCAGCACCGAGGTAAGGCT-3′, reverse 5′-TTACGGAGG GGAAAGTCATCA-3′; *Hdac6*, forward 5′-ACCACCTCTCTGGAGGCTT-3′, reverse 5′-TGG GGTCACAGCATAAAATACATC-3′; *Hdac7*, forward 5′-AACTTCGGCAACTTCTCAATAA-3′, reverse 5′-GGGTGTGCTGCTACTACTGGG-3′; *Hdac8*, forward 5′-ATGGCCACCTTCCA CACTG-3′, reverse 5′-CTTTGCATGATGCCACCCTC-3′; *Hdac9*, forward 5′-ATGCCTGTGG TGGATCCTGT-3′, reverse 5′-AGAGGAGGAAGCTGCTGCTC-3′; *Hdac10*, forward 5′-TGG CACCGCTATGAGCAT-3′, reverse 5′-GACACCAGCACCAACTCAGG-3′; *Hdac11*, forward 5′-GGCAGCGAAGGTAACATCTA-3′, reverse 5′-CACATCCTCTTACCCCTGTG-3′; *Gja1*, forward 5′-GAGAGCCCGAACTCTCCTTT-3′, reverse5′-TGGAGTAGGCTTGGACCTTG-3′; *Gja5*, forward 5′-CAGAGCCTGAAGAAGCCAAC-3′, reverse 5′-GCAACCAGGCTGAATG GTAT-3′; *Cdhc2*, forward 5′-TATGTGATGACGGTCACTGC-3′, reverse 5′-GAAAGGCCAT AAGTGGGATT-3′; and *Gapdh*, forward 5′-TGCCACTCAGAAGACTGTGG-3′, reverse 5′-AGGAATGGGAGTTGCTGTTG-3′.

### WESTERN BLOTTING

Ventricular myocytes were cultured at high density in 35 mm culture dishes for 4 days in 3 ml of BrDU/M199 media, harvested, and lysed with 1% Triton X-100 extraction buffer (50 mM Tris pH 8.0, 150 mM NaCl, 0.02% sodium azide, 1.0 mM phenylmethanesulfonylfluoride (PMSF), 1 μg/ml aprotinin, 1% Triton X-100, 1 mM Na_3_VO_4_, 50 mM sodium fluoride (NaF) with protease inhibitors (Roche). One dish from each primary culture served as a control sample and a second dish was treated with either TSA or VOR for 24 h prior to harvesting. Sonicated samples (three 30-s pulses) were incubated on ice for 30 min, centrifuged at 14,000 rpm (10 min at 4°C), transferred to new tubes, and protein concentrations were measured using the coomassie blue protein assay (Bio-Rad). Fifteen micrograms of total protein/sample was heated (55°C) and loaded onto an SDS-PAGE (sodium dodecyl sulfate polyacrylamide gel electrophoresis) gel and electrophoresed for 90 min at 110 V in 4× NuPAGE sampling buffer and 10× NuPAGE reducing buffer (Invitrogen). The protein gels were transferred onto polyvinylidene difluoride (PVDF) membranes for 90 min at 4°C (110 V), blocked with 5% non-fat milk for 1 h at room temperature, and incubated overnight at 4°C with Cx43, Ncad, α-tubulin, or α-actin primary antibodies in phosphate-buffered saline (PBS) with Tween 20 (PBS-T) with 5% non-fat milk. The membranes were washed 5 min × 4 with PBS-T, incubated with horseradish peroxidase (HRP)-labeled secondary antibody (1:5000) at room temperature in PBS-T with 5% non-fat milk for 30 min, washed again 5 min × 4 with PBS-T, and developed using the ECL^TM^ Western Blot Detection Reagents (Bio-Rad). The image was taken by exposuring light sensitive films (Midsci) to the PVDF membrane and developing the films using the Auto-developer (Kodak) in a dark room. The density of the bands was quantified using ImageJ. Primary antibodies used in this study include rabbit anti-Cx43 (Chemicon), mouse anti-Cx43 (Zymed), mouse anti-Cx40 (Zymed), mouse anti-α-tubulin (Sigma), rabbit anti-acetylated-α-tubulin (Enzo), mouse anti-α-actin antibody (Sigma), rabbit anti-acetylated lysine antibody (Abcam), and Ncad (Sigma). pSer-specific Cx43 antibodies were produced as previously described including rabbit anti-pS255 (Santa Cruz; Sirnes et al., 2009), rabbit anti-pS262 (Santa Cruz; Srisakuldee et al., 2006), rabbit anti-pS279/282 (Santa Cruz; Arnold et al., 2005; Solan and Lampe, 2008), rabbit anti-pS325/328/330 (Lampe et al., 2006), rabbit anti-pS365 (Solan et al., 2007), rabbit anti-pS368 (R&D; Solan et al., 2007), and rabbit anti-pS373 (P. D. Lampe, personal communication, manuscript in preparation).

### IMMUNOSTAINING, IMAGING, AND ANALYSIS

Ventricular myocytes were cultured at high density on 10 μg/ml fibronectin-coated 18 mm diameter glass coverslips in 12-well plates with 1 ml of BrDU/M199 media for 4 days. Control and TSA- or VOR-treated myocyte cultures were fixed with 4% paraformaldehyde, permeabilized with 1% Triton X-100 in PBS, and blocked by the addition of 2% goat serum at room temperature for 15 min. Primary mouse anti-Cx43 antibody (Invitrogen) was diluted 1:500 in 2% goat serum/1% Triton X-100/PBS, 250 μl added to each coverslip, and incubated overnight at 4°C according to previously published procedures (Lin et al., 2010). Coverslips were washed three times with PBS, incubated in the dark with Alexa Fluor546 goat anti-mouse secondary antibodies (1:1500 dilution) for 3 h at room temperature, washed three times with PBS, stained with 4′,6-diamidino-2-phenylindole (DAPI) for 5–10 min, rinsed twice with PBS and finally with distilled, deionized water and mounted onto glass slides with the Prolong Gold antifade medium (Invitrogen).

Confocal fluorescence micrographs for the TSA experiments were acquired using the Zeiss LSM 510 META confocal microscope core facility and viewed using the Zeiss LSM Image Browser V3.5 software. Confocal fluorescence micrographs for the VOR experiments were acquired using the Perkin-Elmer UltraView Vox dual spinning disk confocal microscope facility located in the Cell and Developmental Biology Department of State University of New York Upstate Medical University. Optical sections were acquired with 0.5 μm resolution (≈ 1λ since fluorophore is Alexa Fluor546 (nm) and one section from the center of the Z-stack representing the highest GJ area was exported as a TIF file, imported into ImageJ, converted to 8-bit red color, background subtracted using the fluorescence intensity histogram (10–25%, average ≈ 10%), and converted to black-on-white bitmaps of Cx43 immunofluorescent regions in ImageJ based on the methods of Hunter et al. (2005). Only confluent ventricular cell monolayer regions were imaged and five regions per coverslip were sampled. Each experiment was repeated three times, resulting in 15 analyzed images per [TSA] or [VOR] experiment. GJ area was expressed as the % of immunolocalized Cx43 area per total area of each image and each experiment was performed on cultured glass coverslips treated with 0, 20, 50, or 100 nM TSA or 0, 0.2, 0.5, 1.0, or 5.0 μM VOR prepared from the same ventricular myocyte primary culture.

### CHROMATIN IMMUNOPRECIPITATION ASSAY

Ventricular myocytes were seeded at high density in 35 mm culture dishes, cultured for 4 days in BrDU/M199 media, treated overnight with 2 μM VOR, fixed with 1% formaldehyde, collected and stored in -80°C for chromatin immunoprecipitation (ChIP) assay. ChIP assays were carried out using the ChIP Assay kit (Millipore Catalog #17-295) according to the manufactures procedures. The cells were lysed with SDS lysis buffer (1% SDS, 10 mM ethylenediaminetetraacetic acid (EDTA), 50 mM Tris, 1 mM PMSF, 1 μg/ml aprotinin, and 1 μg/ml pepstatin A, pH 8.1, sonicated to shear the chromatin DNA to 400–1000 bp, diluted 10-fold in ChIP dilution buffer (0.01% SDS, 1.1% Triton X-100, 1.2 mM EDTA, 16.7 mM Tris–HCl, 167 mM NaCl, 1 mM PMSF, 1 μg/ml aprotinin, and 1 μg/ml pepstatin A, pH 8.1), and pre-cleared with 50 μl protein A agarose/salmon sperm DNA (supplied with the kit) for 30 min at 4°C. The pre-cleared cell lysis was immunoprecipitated with 10 μg of rabbit anti-HDAC1 antibody (Enzo Life Sciences), rabbit anti-HDAC2 antibody (Enzo Life Sciences), rabbit anti-Sp1 antibody (Santa Cruz), rabbit anti-RNA Pol II antibody (Santa Cruz), or normal rabbit IgG (Sigma) overnight (4°C). 50 μl of protein A agarose/salmon sperm DNA was added the next morning and incubated for 1 h at 4°C. The agarose was pelleted by centrifugation at 1000 rpm for 1 min, the pellets were washed with low salt buffer (0.1% SDS, 1% Triton X-100, 2 mM EDTA, 20 mM Tris–HCl, 150 mM NaCl pH 8.1), high salt buffer (0.1% SDS, 1% Triton X-100, 2 mM EDTA, 20 mM Tris–HCl, 500 mM NaCl pH 8.1), LiCl buffer (0.25 M LiCl, 1% IGEPAL-CA630, 1% deoxycholic acid, 1 mM EDTA, 10 mM Tris, pH 8.1), and TE buffer (1 mM EDTA, 10 mM Tris, pH 8.0). The agarose/protein/DNA complex was eluted with 250 μl elution buffer (0.1 M NaHCO_3_, 1% SDS), at room temperature for 15 min and the elution step was repeated once. Twenty microliters of 5 mol/l NaCl was added to the 500 μl elution solution, and heated for 4 h at 65°C to reverse protein–DNA crosslinks. Ten microliters of 0.5 M EDTA, 20 μl of 1 M Tris–HCl (pH 6.5), and 2 μl of 10 mg/ml proteinase K was added to the elution solution and incubated 1 h at 45°C. The DNA in the elution was purified with GENECLEAN^®^ II Kit (MP) and tested by PCR amplification for 26 cycles. Primers specific for the mouse Cx43 promoter were: primer set-1 of 5′-TCCCATGCCCACCGCCTCTT-3′ (sense) and 5′-TGCGGGGCTGTGACTCCTCA-3′ (antisense) corresponding to nucleotide positions -511 to -492 bp and -225 to -206 bp; primer set-2 of 5′-TGAGGAGTCACAGCCCCGCA-3′ (sense) and 5′-TCCCTCACGCCTTTCCCCCA-3′ (antisense) corresponding to nucleotide positions -225 to -206 bp and +65 to +84 bp, with respect to the transcription start site of Cx43.

### ELECTROPHYSIOLOGY

Dual whole cell patch clamp experiments were performed on isolated ventricular myocyte cell pairs as previously described (Lin et al., 2005, 2008a, 2010). The initial GJ conductance (*g*_j_) was measured immediately upon establishment of the dual whole cell patch clamp configuration from the linear slope portion of a 2-s -100 to +100 mV transjunctional voltage (*V*_j_) ramp. Ventricular GJ channel activity was recorded during 30-s *V*_j_ pulses ranging from ±20 to ±60 mV. All points current amplitude histograms were produced from each junctional current (*I*_j_) recording and fitted with Gaussian distributions in Origin7.5 to determine the mean GJ channel current (*i*_j_) amplitudes. The linear slope of the cumulative *i*_j_–*V*_j_ relationship from three experiments was used to calculate the single GJ channel conductance (γ_j_). Steady state *V*_j_-dependent inactivation (increasing *V*_j_) and recovery (decreasing *V*_j_) normalized junctional conductance–voltage (*G*_j_–*V*_j_) curves were obtained using a 200 ms/mV, ±120 mV voltage ramp protocol. Quantitative junctional voltage series resistance errors were corrected for each patch electrode by the expression (Veenstra, 2001):

(1) $$\begin{array}{c} g_{\text{j}}=\frac{-\Delta I_{2}}{V_{1}-(I_{1}\cdot R_{\text{el}1})-V_{2}+(I_{2}\cdot R_{\text{el}2})}\,,\; \end{array}$$

where *V*_1_ and *V*_2_ are the command potentials applied to cells 1 and 2, *I*_1_ and *I*_2_ are the corresponding whole cell currents, *R*_el1_ and *R*_el2_ are the corresponding whole cell patch electrode resistances, and -Δ*I*_2_ is the change in *I*_2_ recorded with constant *V*_2_ during a voltage step applied to *V*_1_ (Δ*V*_1_; Veenstra, 2001). *I*_j_ is assumed to originate from the Δ*V*_1_ pulse with the same polarity, therefore *I*_j_ = *I*_2_ - Δ*I*_2_. The resultant inactivation and recovery *G*_j_–*V*_j_ curves were fit with the Boltzmann equation:

(2) $$\begin{array}{c} G_{\text{j}}^{\text{ss}}=[\frac{G_{\text{max}}^{\text{ss}}\cdot [\text{exp (}A\cdot (V_{j}-V_{1/2}))]\;+\;G_{\text{min}}^{\text{ss}}}{1+[\text{exp (}A\cdot (V_{j}-V_{1/2}))]}], \end{array}$$

where $G_{\text{j}}^{\text{ss}}$ = *g*_j_/*g*_j,max_, $G_{\text{max}}^{\text{ss}}$ = the maximum value of *g*_j_/*g*_j,max_ = 1, $G_{\text{min}}^{\text{ss}}$ = the minimum value of *g*_j_/*g*_j,max_, *A* = the slope factor for the Boltzmann curve (= zF/RT at 20°C), and *V*_½_ = the half-inactivation voltage. The actual *g*_j,max_ value is determined from the linear slope conductance of the *I*_j_–*V*_j_ relationship for each experiment. The *I*_j_–*V*_j_ relationship is typically linear between ±10 and ±25 mV. Curve-fitting procedures were performed using Clampfit software (pClamp version 8.2, Axon Instruments, Inc.) and final graphs were prepared using Origin version 7.5 software (OriginLab Corporation, Northampton, MA, USA). Tetrodotoxin (30 μM TTX, Sigma) was added to the bath saline (mM: NaCl 142, KCl 1.3; CsCl 4, TEACl 2, MgSO_4_ 0.8, NaH_2_PO_4_ 0.9, CaCl_2_ 1.8, dextrose 5.5, 4-(2-hydroxyethyl)-1-piperazineethanesulfonic acid (HEPES) 10, pH 7.4 with 1 N NaOH) of each dish to prevent activation of the sodium current during the dual whole cell patch clamp experiments. Patch pipettes measuring 4–5 MΩ before patch break were filled with a KCl internal pipette solution (IPS KCl, in mM: KCl, 140; MgCl_2_, 1.0; CaCl_2_, 3.0; 1,2 bis(*o*-amino-phenoxy)ethane-*N,N,N′,N′*-tetra acetic acid (BAPTA), 5.0; HEPES, 25; pH titrated to 7.4 using 1 N KOH). The osmolarity of both external and internal solutions was adjusted to 310 mOsm/l.

### STATISTICAL ANALYSIS

One-way ANOVA was performed on multiple experimental datasets in Origin7.5 with the Levene's test for equal variance and the Bonferroni means comparison test. Actual *p* values are shown in the figures when statistically significant (*p* < 0.05)

## RESULTS

### HDAC EXPRESSION AND INHIBITION IN CULTURED VENTRICULAR MYOCYTES

The relative expression of all 11 HDACs in neonatal mouse ventricular myocyte cultures was examined by RT-PCR. HDAC mRNA levels ranged from 1 to 16% relative to Cx43 mRNA levels (**Figure 1A**). Cx40 mRNA levels were 1.6 ± 0.7% (mean ± SEM) relative to Cx43, indicative of contributions from the ventricular conduction system and coronary endothelial cells. The same relative HDAC mRNA expression pattern was found in atrial cardiomyocyte cultures wherein the Cx40 mRNA level was 38.4 ± 5.9%, consistent with the contribution of Cx40 to atrial myocardial GJs (**Figure 1A**; Lin et al., 2010). TSA, the prototypical hydroxamic acid pan-HDACI, suppressed ventricular HDAC activity with apparent inhibitory equilibrium constants (*K*_I_s) of 12 and 174 nM (**Figure 1B**). HeLa cells exogenously expressing Cx43 (stable HeLa-Cx43 cells) exhibited a single *K*_I_ of 510 nM. VOR, by comparison, inhibited ventricular cardiac HDAC activity with approximately 10-fold higher *K*_I_s of 186 and 1440 nM (**Figure 1C**). HeLa-Cx43 cell inhibition by VOR also exhibited a dual affinity profile with *K*_I_s of 300 nM and 2 μM. Ventricular cardiomyocyte total HDAC activity declined to a minimum of 2–6% with TSA or VOR inhibition.

### TRICHOSTATIN A AND VORINOSTAT AFFECT CX43 MRNA AND PROTEIN EXPRESSION

**FIGURE 2:**
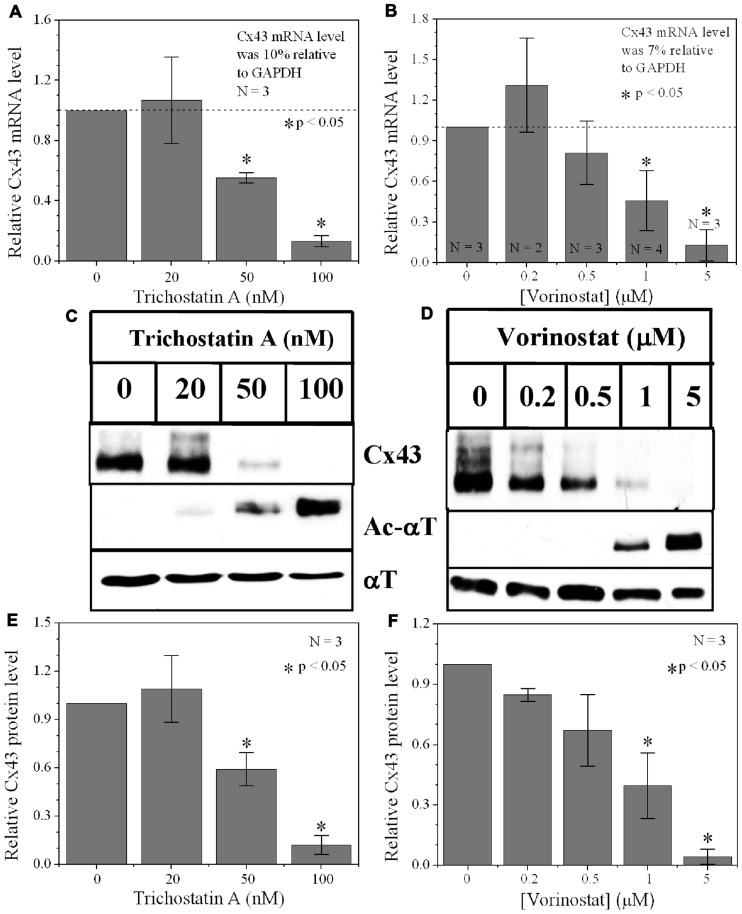
**Dose-dependent alteration of Cx43 expression by pan-HDACI**. **(A,B)** Real-time PCR results of Cx43 mRNA expression levels after overnight inhibition by increasing [TSA] **(A)** and [VOR] **(B)** relative to control (untreated) values. Experiments were performed in triplicate and the lowest dose of both pan-HDACI produced an insignificant increase in average Cx43 mRNA levels and a significant reduction in Cx43 mRNA levels at the highest doses. **(C,D)** Representative Cx43 western blots of ventricular cell lysates from TSA **(C)** or VOR **(D)** treated cultures. **(E,F)** Statistical analysis of the protein densitometry scans from three experiments reveal significant dose-dependent reductions in Cx43 protein content. Cx43 protein levels were normalized to a control sample from each experiment with α-tubulin (αT) expression used as an internal control and acetylated α-tubulin (Ac-αT) as a positive indicator for HDAC inhibition.

**FIGURE 3:**
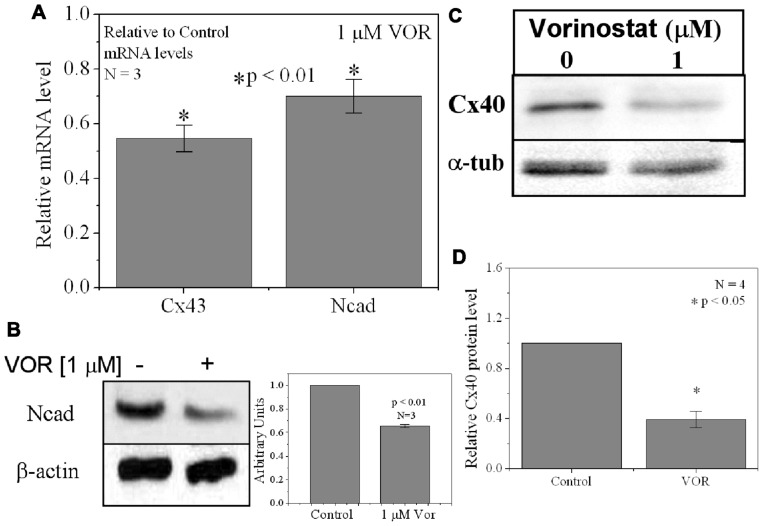
**Reduction of Cx40 and N-cadherin expression by HDACI**. **(A)** Overnight (18 h) treatment with 1 μM VOR significantly reduced Cx43 and N-cadherin mRNA levels by 45 ± 5 and 30 ± 6%, respectively. Experimental mean values were statistically different from control values (*p*-value < 0.05, one-way ANOVA). **(B)** Immunoblots were also performed for Ncad and β-actin (internal control). Total Ncad levels from three experiments were decreased by 1 μM VOR relative to control (untreated) samples. **(C)** Representative Cx40 western blots of atrial cell lysates from control or 1 μM VOR-treated cultures. **(D)** Statistical analysis of the protein densitometry scans from four experiments reveals a significant reduction in Cx40 protein content. Cx40 protein levels were normalized to a control sample from each experiment with α-tubulin expression used as an internal control and acetylated α-tubulin (Ac-αT) as a positive indicator for HDAC inhibition.

**FIGURE 4:**
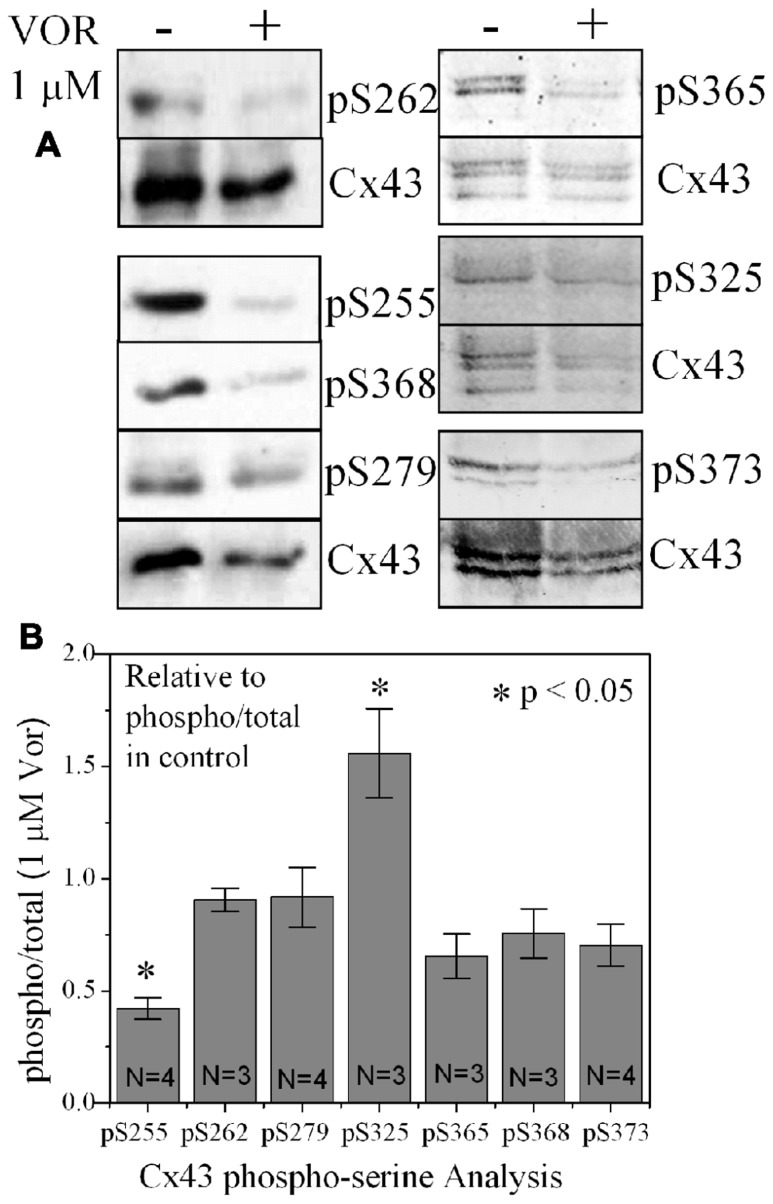
**Alteration of Cx43 phosphorylation state by vorinostat**. **(A)** Representative western blot of control and 1 μM VOR-treated ventricular myocyte lysates detected with Cx43 phospho-serine (pSer) specific anti-pS255, anti-pS262, anti-pS279/282, anti-pS368, anti-pS325/328/330, anti-pS365, and anti-pS373 antibodies. The total Cx43 was also immunoblotted with an anti-Cx43 carboxyl-terminal antibody that recognizes all forms of Cx43. **(B)** Cx43 phospho-serine pS255, pS262, pS279/282, pS368, pS325/328/330, pS365, and pS373 levels were compared by densitometry to the total Cx43 levels in control and 1 μM VOR-treated samples to generate the phospho/total Cx43 ratios. The VOR-treated phospho/total Cx43 ratios are plotted relative to control Cx43 phospho/total ratio.

Since protein acetylation is known to alter gene expression, we examined the effect of HDAC inhibition by TSA and VOR on Cx43 mRNA and protein expression levels by RT-PCR and western blot analyses. The lowest doses of TSA and VOR tested did not significantly change Cx43 mRNA expression, though further increases in [TSA] or [VOR] produced a progressive decline in Cx43 mRNA levels to a minimum of 13% relative to untreated control values (**Figures 2A,B**). One micromolar VOR reduced the Cx43 mRNA expression levels by 54%. The transcriptional effects of HDACI by TSA or VOR were verified at the translational level by western blot analysis of Cx43 (**Figures 2C–F**). Acetylated α-tubulin levels increased with both TSA and VOR. Densitometry scans of three HDACI western blot experiments confirm a gradual dose-dependent reduction in the amount of Cx43 protein to a minimum of 5–12% of control levels by TSA and VOR. The recommended therapeutic dose of 1 μM VOR reduced Cx43 protein levels by an average of 60 ± 3%. Ncad mRNA and protein levels were reduced by ≈30% (**Figures 3A,B**; Kerr et al., 2010). This dose of VOR also reduced atrial cardiomyocyte Cx40 protein expression by 64 ± 6% (**Figures 3C,D**). The Cx43 and Cx40 levels relative to the α-tubulin loading controls were similar, 1.26 ± 0.30 for Cx43 (*n* = 3, mean ± SEM) and 1.26 ± 0.26 for Cx40 (*n* = 4). We further examined whether ventricular HDAC inhibition by 1 μM VOR could affect the phosphorylation state of Cx43 using custom and commercially available pSer-specific Cx43 antibodies. Western blot analysis of seven identified Cx43 pSer sites revealed altered protein kinase-dependent Cx43 phosphorylation content relative to control levels. Since total Cx43 was downregulated by HDACI treatment, the phospho/total Cx43 ratios of the treated samples were normalized to the phospho/total ratios of the control samples to analyze the changes of pSer levels by VOR. The results indicated additional downregulation of phosphorylation at S255 (pS255) by 57.9 ± 4.9%. Conversely, the phosphorylation of S325/328/330 was upregulated by 48.5 ± 19.8% in presence of 1 μM VOR. The pS262, pS279/282, pS365, pS368, and pS373 levels were not significantly altered (**Figures 4A,B**).

### HDACI ALTERS PROTEIN BINDING TO THE CX43 PROMOTER REGION

**FIGURE 5:**
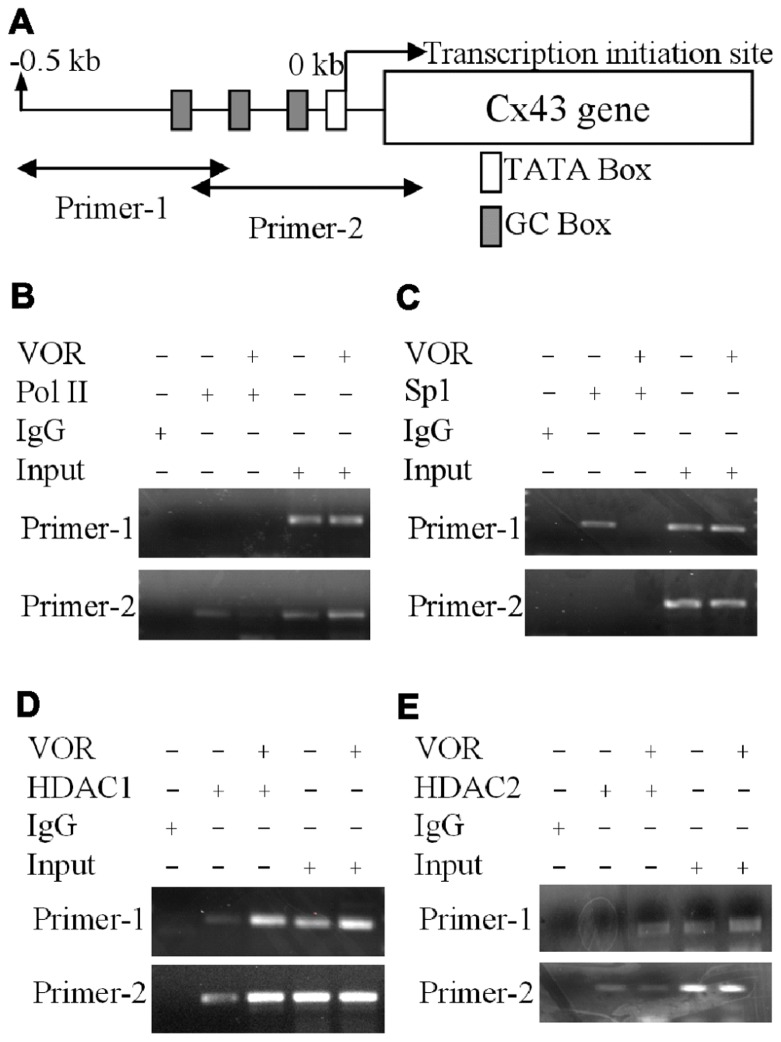
** Alteration of Cx43 promoter associated proteins by vorinostat**. **(A)** Illustration of the Cx43 promoter region up to -0.5 kb from the Cx43 gene (*Gja1*) transcription start site of Cx43 with TATA and GC boxes indicated. Two primer sets were designed from -511 to -206 and -225 to +65 bp of the Cx43 promoter region. **(B,C)** Chromatin immunoprecipitation assays illustrate the reduced binding of RNA polymerase II (RNA Pol II) and specific protein 1 (Sp1) to the promoter region of Cx43 after overnight exposure to 2 μM VOR. **(D,E)** A chromatin immunoprecipitation assay was performed with rabbit IgG and rabbit anti-HDAC1 or anti-HDAC2 antibodies to assess the binding of HDAC1 **(D)** and HDAC2 **(E)** to the promoter region of the Cx43 gene (*Gja1*). Overnight treatment with 2 μM VOR enhanced the association of HDAC1 and HDAC2 to the *Gja1* promoter.

To examine whether VOR induced repression of Cx43 (*Gja1*) gene expression by altering protein binding to the proximal promoter of Cx43 in heart, ChIP assays were performed to examine the association of Sp1 (specificity protein 1) and RNA polymerase II (RNA Pol II) with the *Gja1* promoter. Up to 0.5 kb of the *Gja1* promoter containing one TATA and several GC boxes was examined by PCR using two sets of primers (**Figure 5A**). Primer set-1 specifically amplified the -511 to -206 bp and primer set-2 specifically recognized -225 to +65 bp of the *Gja1* promoter region. The results indicate that 2 μM VOR decreased association of Sp1 and RNA Pol II with the Cx43 promoter (**Figures 5B,C**). *Gja1* promoter association with Sp1 was detected by primer set-1 while only primer set-2 detected RNA Pol II association. The association of HDAC1 and 2 with the *Gja1* promoter was increased by VOR treatment (**Figures 5D,E**). The expression of HDAC1, 2, Sp1, and RNA Pol II were not altered by VOR.

### HDACI DECREASES CX43 GAP JUNCTION AREA

**FIGURE 6:**
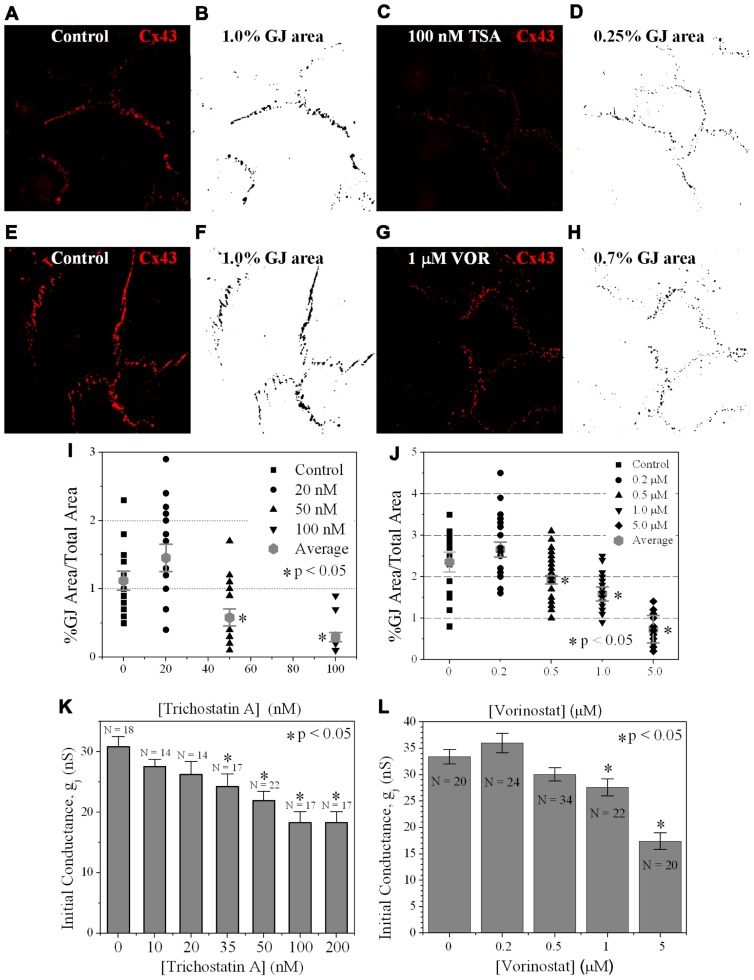
**Measurement of Cx43 GJ area and ventricular electrical coupling**. **(A–H)** Representative confocal images of mouse primary anti-Cx43 and goat anti-mouse Alexa Fluor 546 secondary antibody immunolocalized Cx43 gap junction plaques from 4-day ventricular myocyte cultures **(A,C,E,G)** and their corresponding black-on-white bitmaps of the Cx43-positive pixels **(B,D,F,H)**. The total GJ area was calculated relative to the total image area of the confluent monolayer cardiomyocyte cluster. Five clusters were imaged per coverslip and each experiment was repeated in triplicate for each [TSA] and [VOR]. **(I,J)** Statistical analysis of the average Cx43 GJ area values (±SEM, *n* = 15) at each pan-HDACI concentration revealed a significant decrease in GJ area for [TSA] ≥ 50 nM and [VOR] ≥ 500 nM. **(K,L)** Initial gap junction conductance (*g*_j_) measurements, upon establishment of the dual whole cell patch clamp configuration, from numerous (*n* = 14–34) cell pairs revealed a dose-dependent maximum decrease in ventricular *g*_j_ of 40–50% by pan-HDACI. The decline in ventricular *g*_j_ was statistically significant for [TSA] ≥ 35 nM and [VOR] ≥ 1 μM.

Since TSA and VOR reduced Cx43 mRNA and protein levels, we examined whether HDACI also affected the formation of Cx43 GJs between cultured ventricular myocytes. Cx43 was immunolocalized in cardiomyocytes after culturing on glass coverslips for 3 days and overnight TSA or VOR treatments. Five micrographs of confluent fields from each coverslip were imaged on a confocal microscope and the Cx43 immunolabeled area was quantified using ImageJ (**Figures 6A–H**). Each [TSA] and [VOR] experiment was repeated three times and the maximum % of Cx43 GJ area was tabulated for each HDACI concentration. Again, the lowest dose of TSA or VOR produced a modest increase in Cx43 GJ area followed by a progressive dose-dependent decrease in GJ area to a minimum of 25% relative to control values (**Figures 6I,J**).

### FUNCTIONAL CONSEQUENCES OF HDAC INHIBITION ON VENTRICULAR GAP JUNCTIONS

#### Gap junction conductance

To examine whether the downregulation of Cx43 expression and GJ assembly by pan-HDACI translates into functional alterations of electrical coupling, the effects of TSA and VOR on functional Cx43-mediated GJ electrical coupling was studied in dual whole cell patch experiments of isolated ventricular myocyte cell pairs. The GJ conductance (*g*_j_) was measured at the onset of dual whole patch clamp recordings of junctional current (*I*_j_) in ventricular myocyte cultures treated overnight with TSA or VOR (**Figures 6K,L**). Increasing concentrations of TSA produced a progressive decline in electrical coupling between ventricular cardiomyocytes that achieved statistical significance above 35 nM. VOR produced a slight increase in electrical coupling at 200 nM followed by a gradual decrease in ventricular *g*_j_. The recommended therapeutic dose of 1 μM VOR reduced ventricular *g*_j_ by 20% (*p* < 0.05). Maximal HDAC inhibition by TSA (≥100 nM) or VOR (5 μM) produced significant 40–50% decreases in ventricular *g*_j_.

#### Transjunctional voltage gating

**FIGURE 7:**
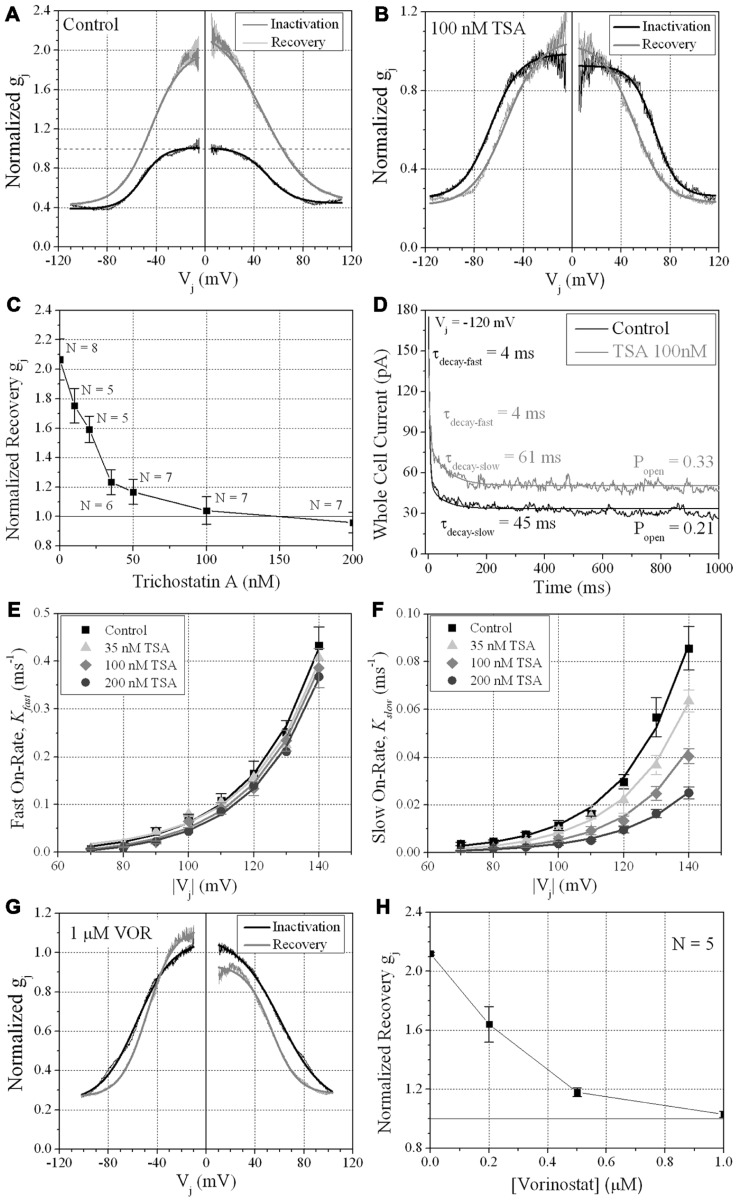
***V*_j_-dependent gating of ventricular gap junctions during HDAC inhibition**. A 200 ms/mV *V*_j_ ramp from 0 to ±120 mV was applied to patch clamped ventricular myocyte pairs and *V*_j_ was returned to 0 mV by ramp reversal to produce the normalized junctional conductance–voltage (*G*_j_–*V*_j_) inactivation and recovery curves. The black line represents the normalized *g*_j_ during the increasing *V*_j_ (inactivation) ramp and the gray line is the *G*_j_–*V*_j_ curve obtained during the decreasing *V*_j_ (recovery) ramp. The facilitated recovery of *g*_j_ was observed as an increase in the normalized *g*_j_ (*G*_j_) of the recovery curve relative to the initial slope *g*_j_ of the ventricular gap junctions during the increasing (inactivation) phase of the *V*_j_ ramp (inactivation curve normalized slope *g*_j_ = 1.0). The smooth black and gray lines are Boltzmann equation fits of the ventricular *G*_j_–*V*_j_ curves obtained from five to six experiments from control myocytes **(A)**, 100 nM TSA-treated myocytes **(B)**, or 1 μM VOR **(G)**. The parameters for the Boltzmann fits of the 100 nM TSA and 1 μM VOR *G*_j_–*V*_j_ inactivation and recovery curves are listed in **Table 1**. **(C)** The slope *G*_j_ of the recovery *G*_j_–*V*_j_ curves was increased in normal ventricular myocytes and abolished by TSA treatments in a dose-dependent manner. **(D)** The inactivation kinetics were determined in control and TSA-treated ventricular myocyte pairs by ensemble averaging the *I*_j_ from 5 to 10 *V*_j_ pulses from -70 to -140 mV. The ensemble averaged *I*_j_ trace was fitted with a second-order decaying function and the fast and slow inactivation rates were calculated from the expression *k*_on_ = (1 - *P*_open_)/τ_decay_. One example is shown for a control (black line) and 100 nM TSA-treated (gray line) myocyte pair in response to a train of -120 mV *V*_j_ pulses. **(E,F)** The fast **(E)** and slow **(F)** on-rates for hypothesized inactivation particles were plotted relative to the absolute value of the *V*_j_ pulses and fitted with first-order exponential increasing functions. The fast inactivation rates were not affected by TSA while the amplitude, but not the *V*_j_-dependence, of the slow inactivation rates were progressively reduced by increasing TSA concentrations. **(G)** The smooth black and gray lines are Boltzmann equation fits of the ventricular *G*_j_–*V*_j_ curves obtained from five 1.0 μM VOR-treated myocyte pairs (see **Table 1**). **(H)** The slope *G*_j_ of the recovery *G*_j_–*V*_j_ curves, increased under normal conditions, was abolished by VOR treatments in a dose-dependent manner.

We previously described the steady state transjunctional voltage (*V*_j_) gating of ventricular GJs using continuous 24 s, ±120 mV *V*_j_ staircase and reported the phenomenon of a higher slope *g*_j_ during the return (declining) phase of the *V*_j_ staircase termed facilitation (**Figure 7A**; Lin et al., 2005). TSA reduced the amplitude of this *increased* *G*_j_ during the recovery phase in a dose-dependent manner, eliminating any increase in recovery *G*_j_ when [TSA] ≥ 100 nM (**Figures 7B,C**). VOR also abolished *G*_j_ facilitation in a dose-dependent manner (**Figures 7G,H**). TSA and VOR also slightly altered the *V*_j_-dependent inactivation properties of ventricular GJs. TSA increased the half-inactivation voltage (*V*_½_) of the inactivation and recovery *G*_j_–*V*_j_ curves by 10–15 mV with a slight reduction in the *V*_j_-insensitive *G*_j_ component (*G*_min_) from 0.40 to 0.25 (**Table 1**). VOR also shifted the *V*_½_ values outward by 5–10 mV. The kinetics of time-dependent inactivation were examined for varying [TSA] by exponentially fitting the *I*_j_ curves to determine the fast and slow decay time constants (τ_fast_ and τ_slow_; **Figures 7D–F**). The fast and slow inactivation on-rates (*K*_fast_ and *K*_slow_) were calculated using the equation *K* = (1 - *P*_open_)/τ_decay_. The *K*_fast_ and *K*_slow_ values increased exponentially every 20.9 ± 1.4 or 20.0 ± 1.2 mV, respectively, and were not altered by TSA. However, the amplitude of the *K*_slow_ inactivation component decreased progressively with increasing TSA concentrations.

**Table 1:** *V*_j_-gating properties of ventricular gap junctions.

Parameter* Connexin	*G*_j,max_ (-*V*_j_)	*G*_j,min_ (-*V*_j_)	*V*_½_ (mV)	*z (q)*	*G*_j,max_ (+*V*_j_)	*G*_j,min_ (+*V*_j_)	*V*_½_ (mV)	*z (q)*	Correlation coefficient
Inactivation control (*n* = 5)	1.011 ± 0.002	0.387 ± 0.001	-51.4 ± 0.1	-2.80 ± 0.03	1.011 ± 0.002	0.445 ± 0.001	+50.9 ± 0.1	+2.28 ± 0.02	0.98 0.97
Inactivation TSA (*n* = 6)	0.987 ± 0.001	0.252 ± 0.002	-66.7 ± 0.1	-2.13 ± 0.02	0.926 ± 0.002	0.262 ± 0.002	+67.5 ± 0.1	+2.73 ± 0.04	0.97 0.96
Inactivation VOR (*n* = 5)	1.064 ± 0.002	0.240 ± 0.003	-56.5 ± 0.1	-1.71 ± 0.02	1.088 ± 0.002	0.216 ± 0.003	+60.9 ± 0.1	+1.40 ± 0.01	0.98 0.99
Recovery control (*n* = 5)	1.995 ± 0.005	0.427 ± 0.002	-44.3 ± 0.1	-1.97 ± 0.02	2.242 ± 0.009	0.451 ± 0.005	+47.9 ± 0.2	+1.38 ± 0.02	0.98 0.98
Recovery TSA (*n* = 6)	1.058 ± 0.003	0.216 ± 0.002	-54.4 ± 0.2	-1.81 ± 0.02	1.037 ± 0.003	0.230 ± 0.002	+50.7 ± 0.1	+2.00 ± 0.02	0.96 0.96
Recovery VOR (*n* = 5)	1.127 ± 0.002	0.267 ± 0.001	-48.1 ± 0.1	-2.30 ± 0.02	0.936 ± 0.002	0.281 ± 0.002	+53.5 ± 0.1	+2.36 ± 0.02	0.98 0.97

*Parameters were determined by curve-fitting the *G*_j_–*V*_j_ curves with Eq. 2.

#### Single gap junction channel conductance

**FIGURE 8:**
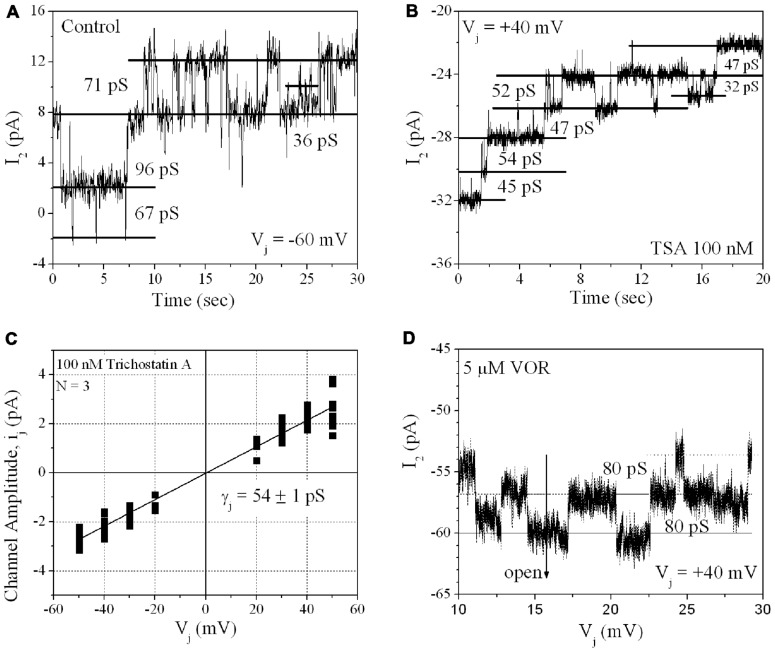
**Single gap junction channel conductance (γ_j_) of HDACI-treated ventricular myocytes**. **(A)** A representative example of single channel open–closed events during an *I*_j_ recording in response to a +60 mV, 30 s *V*_j_ pulse applied to a pair of primary cultured ventricular myocytes. Note the activity of low (30–50 pS), intermediate (60–80 pS), and high (90–110 pS) γ_j_ states typical of Cx43 GJ channel activity. **(B)** An example of GJ channel activity recorded from a pair of ventricular myocytes treated with 100 nM TSA during a -40 mV, 30 s *V*_j_ pulse. Only low (30–50 pS) γ_j_ channel activity was observed in this experiment. **(C)** Single gap junction channel current (*i*_j_) amplitudes were measured from all point histograms for every *V*_j_ pulse obtained from three different 100 nM TSA-treated ventricular myocyte cell pairs. Linear regression analysis of the composite *i*_j_–*V*_j_ relationship had a slope conductance of 54 ± 1 pS. **(D)** Representative example of single channel open–closed events during an *I*_j_ recording in response to a +40 mV, 30 s *V*_j_ pulse applied to a pair of primary cultured ventricular myocytes treated with 5 μM VOR. In two 5 μM VOR-treated ventricular myocyte pairs, only intermediate 80 pS GJ channel activity was observed, consistent with the intermediate γ_j_ state of Cx43

From twenty 100-nM TSA ventricular cell pair recordings, single GJ channel currents could be resolved in only three poorly coupled ventricular myocyte cell pairs. GJ channel current (*i*_j_) amplitudes corresponding to low (30–50 pS), intermediate (60–80 pS), and high (90–110 pS) channel conductance (γ_j_) states were observed in untreated cardiomyocyte cell pairs, consistent with previous observations for Cx43 and ventricular GJ channels (Moreno et al., 1994). In the three 100-nM TSA experiments, ventricular γ_j_ was reduced to 54 pS, corresponding to only the low γ_j_ state of Cx43 GJ channels. The 80 pS state was still evident in the presence of 5 μM VOR (**Figure 8**).

## DISCUSSION

Our results show that the atrial and ventricular myocardial HDAC expression pattern is similar. Therefore, any differential effects of a particular HDACI on atrial or ventricular excitability and contractility will depend primarily on the expression of ion channels, transporter, junctional, and contractile proteins unique to the specialized regions of the mammalian heart. The cell-based HDAC activity assay also indicates that VOR inhibits total myocardial HDAC activity with two apparent affinities of approximately 200 nM and 1.4 μM (**Figure 1C**). The nanomolar higher capacity site falls within the recommended serum therapeutic range for VOR of í1 μM (400 mg/day) and likely corresponds to inhibition of the class I HDACs (HDAC1–3 and 8) and the class IIb HDAC6 (Bradner et al., 2010; Kerr et al., 2010). This is important because the reductions in ventricular Cx43 expression and electrical coupling observed in our experiments are not significant until the concentration of VOR meets or exceeds this 1 μM therapeutic value. This dose of VOR lowered Ncad protein content by 33%, Cx43 protein content by approximately 50%, GJ area by 33%, and ventricular *g*_j_ by less than 20% (**Figures 2, 3**, and **6**). The significant reduction in Ncad and functional Cx43 GJ expression may result from occupying the micromolar affinity HDAC site, which could correlate with inhibition of the class IIa HDACs (HDAC4, 5, 7, and 9) since the *K*_I_ of VOR for these HDACs is nearly 10-fold higher (Bradner et al., 2010). We intend to study the differential effects of specific class I, IIb, and IIa HDAC inhibition on myocardial expression and function using class-selective HDACIs like MS-275 or MGCD-0103, tubastatin, and MC-1568, respectively (Mai et al., 2005; Rasheed et al., 2007; Bradner et al., 2010; Butler et al., 2010).

These reductions in cardiac intercalated disk adhesion and communicating junctions may not affect conduction velocity or promote arrhythmias since a 50% global loss of Cx43 content minimally alters myocardial conduction velocity or the incidence of sustained ventricular tachycardias (Danik et al., 2004). However, heterogeneous loss of>80% of Cx43 expression leads to ventricular systolic dysfunction and increased susceptibility to lethal ventricular arrhythmias (Gutstein et al., 2001). Chronic heart failure (CHF) and myocardial infarction (MI) also reduces Cx43 content and remodels GJ connections, potentially predisposing the myocardium to reentrant arrhythmias (Luke and Saffitz, 1991; Kostin et al., 2003). There are no reports of QT interval prolongation or cardiac arrhythmias occurring with VOR (Rasheed et al., 2007), even with single dose administration of twice (i.e., 800 mg/day) the normal therapeutic dose of VOR in 24 patients with advanced malignancies (Munster et al., 2009). QT interval prolongation and associated risk for torsades de pointes (TdP) ventricular tachyarrhythmias were reported in clinical trials with two hydroxamic acid-derived HDACIs, LAQ-824 and LBH-589 (Rasheed et al., 2007). Romidepsin, a tricyclic peptide HDACI, has resulted in corrected QT (QTc) interval prolongation and sudden cardiac death from probable fatal ventricular arrhythmias (Shah et al., 2006; Rasheed et al., 2007). The mechanistic basis for these cardiac arrhythmias remains essentially unknown since preliminary studies suggest that human ether-a-go-go potassium channel (Kv11.1) protein (HERG) blockade develops only in the supra micromolar range for LBH-589 and VOR (Giles et al., 2006; Kerr et al., 2010).

In general, HDACI is thought to improve Cx43 GJ intercellular communication (GJIC) between cancer cells (Ogawa et al., 2005; Hernandez et al., 2006). However, HDACIs do not necessarily increase connexin expression and GJIC, owing to different HDACI activities and fundamental differences between immortalized cancer cell lines and primary cell cultures (e.g., hepatocytes, cardiomyocytes; Vinken et al., 2007). The TSA-induced increased transcription of Cx43 (*Gja1*) gene expression requires positive cooperation between Ap1 and Sp1 elements and is associated with hyperacetylated HDAC4 within the 2.4 kb gene promoter region (Hernandez et al., 2006). We analyzed the proximal 500 bp of the *Gja1* gene promoter sequence via ChIP assay (**Figure 5**), inclusive of the Sp1 and four Ap1 sites, and found decreased Sp1 and RNA Pol II and increased HDAC1 and 2 association within this promoter region in the presence of 2 μM VOR. Lower doses of VOR (e.g., 200 nM) were not tested. The altered RNA Pol II and HDAC1 and 2 associations with the *Gja1* gene promoter sequence were not previously described. The HAT P300/CBP associated factor (PCAF), and HDAC3, 4, and 5 reportedly co-localize with Cx43 in the cytosol (Colussi et al., 2011). Precise control of *Gja1* gene transcription by HDAC and HDACI activities requires further investigation using class-specific HDACIs and/or HDAC gene knockout or RNAi knockdown strategies. Again, class-specific HDAC inhibition with second generation HDACIs such as MGCD-0103 or MS-275, tubastatin, and MC-1568 will further delineate the effects of HDACI on *Gja1* gene expression. Preliminary results from our laboratory with sodium phenylbutyrate, a low affinity HDACI with class I/IIa activities, conversely showed an increase in Cx43 protein expression (data not shown). Mice with germline deletion of the class IIa/b HDACs 4–10 are viable while class I HDAC1–3 knockout mice are embryonic lethal and require conditional knockout strategies to be studied further. The impact of pan- and class-selective HDACI or HDAC gene deletion/knockdown on connexin expression should be studied in primary tissues to understand the effects of these emerging HDACI clinical therapies on normal physiology (e.g., cardiac electrophysiology) in addition to the therapeutics effects under pathophysiological conditions in cancerous and other diseased tissues.

Colussi et al. (2010, 2011) did not directly compare the Cx43 expression levels in wt mouse hearts treated with VOR for 96 h although Cx43 levels were reportedly unchanged in wt or mdx mouse hearts. Our *in vitro* results indicate that TSA and VOR first produce a negligible change in Cx43 expression and function followed by a progressive diminution of Cx43 (and Cx40) expression and GJIC with increasing concentrations of pan-HDACI. Increased protein acetylation associated with the mdx mouse or induced by HDACI treatment of wt mice correlated with Cx43 dissociation from cardiac intercalated disk proteins (e.g., ZO-1, Ncad) and lateralization (Colussi et al., 2010, 2011). Cx43 lateralization occurs during ischemia and is associated with dephosphorylation of Cx43, particularly S325/328/330 and S364/365 sites (Lampe et al., 2006; Solan et al., 2007; Solan and Lampe, 2009), and is thought to represent functional GJ downregulation. Colussi et al. (2011) demonstrated that Cx43 is acetylated and that c-Src-dependent Y265 phosphorylation is increased while S255 and S262 phosphorylation of Cx43 is decreased in mdx mouse hearts. They did not examine the effects of VOR on wt hearts. Phosphorylation of Cx43 S262 has been associated with p34^cdc42^, protein kinase C (PKC), mitogen-activated protein kinase (MAPK), and v-Src kinase activity (Solan and Lampe, 2005). Our results indicate that VOR increases phosphorylated S325/328/330 and decreases phosphorylated S255 content while decreasing total ventricular Cx43 expression (**Figure 4**). Cx43 Y265 phosphorylation was not examined in our study and should be considered since Y265, S255, S262, and S368 phosphorylation downregulate while S325/328/330 and S364/365 phosphorylation increase Cx43 GJ function (Lampe et al., 2006; Solan et al., 2007; Solan and Lampe, 2009). Cancer cells may downregulate Cx43 GJIC by reducing expression and/or c-Src and MAPK phosphorylation of Cx43 and altering the phosphorylation state of Cx43 is another mechanism by which HDACI may improve GJIC in malignant tissues.

Direct acetylation may also alter the localization of Cx43 since a triple K-to-Q acetyl-mimetic mutant of Cx43 was predominantly maintained in the cytoplasm whereas the K-to-A acetylation-resistant mutant Cx43 readily formed GJ plaques of unknown function (Colussi et al., 2011). We are presently studying the biophysical GJ gating and channel properties of the putative Cx43 K9, K234, and K264 acetyl-mimetic and -resistant mutations, individually and in combination, to determine the possible effects of Cx43 N^ε^-lysine acetylation on Cx43 GJ formation and function. Our ventricular myocyte patch clamp experiments revealed concentration-dependent decreases in functional GJ coupling and altered *V*_j_-dependent gating properties with TSA and VOR (**Figure 7**; **Table 1**). Ventricular γ_j_ was reduced only by high doses of TSA (**Figure 8**), perhaps because of the higher potency TSA for class IIa inhibition relative to VOR (Bradner et al., 2010). The pan-HDACI-induced decrease in ventricular *g*_j_ probably results from the decreased Cx43 expression and GJ area, which results in a reduced number of GJ channels (*N*), since the channel open probability (*P*_o_) was only slightly affected by the changes in *V*_j_-dependent gating, γ_j_ was reduced only by high doses of TSA, and *g*_j_ = *N*.*P*_o_.γ_j_. We hypothesize that the acetylation-induced changes in γ_j_ and *V*_j_-gating may be due to direct acetylation of Cx43 amino-terminal (K9) and carboxyl-terminal (K234, K264) lysine residues since these cytoplasmic domains have been implicated in these respective GJ channel functions (Moreno et al., 2002; Musa et al., 2004). Post-translational acetylation may affect the Cx43 half-life since lysine ubiquitination is a major degradation pathway for Cx43 GJIC (Kjenseth et al., 2010).

Limitations of the present study include the lack of correlative *in vivo* Cx43 expression and GJ localization results, not normalizing the immunolabeled Cx43 GJ area to cell membrane area by co-staining with fluorescently conjugated wheat germ agglutinin, and no assessment of pan-HDACI effects on myocardial conduction velocity or susceptibility to arrhythmias. Our supply of VOR for these initial functional GJ studies was restricted to *in vitro* use only by the terms of the MTA agreement. Future HDACI studies will include *in vivo* expression and electrocardiogram parameter (e.g., heart rate, QT interval) monitoring. *In vitro* conduction velocity measurements were not possible because we do not presently have access to a multi-electrode array (MEA) system. We hope to provide functional correlates like conduction velocity and the occurrence of spontaneous ventricular contractions, tachycardias, or arrhythmias in future investigations.

In conclusion, the present study demonstrates, for the first time, that pan-HDAC inhibition produces dose-dependent reductions in *Gja1* transcription, Cx43 protein content, Cx43 pSer content, Cx43 GJ area, and functional electrical coupling in normal mammalian ventricular myocardium with lesser effects on *V*_j_-dependent gating and γ_j_ properties. These inhibitory effects on ventricular *g*_j_ correlate with VOR inhibition of myocardial HDAC activity in the micromolar range and suggest inhibition of class IIa HDACs in combination with class I and IIb HDACs may be responsible for these potentially adverse effects. VOR therapy is not associated with the induction of cardiac arrhythmias observed in clinical trials with three different pan-HDACIs, but these results suggest that more potent pan-HDAC inhibitory profiles may be more likely to cause adverse cardiac effects, including reduced myocardial electrical communication, that may predispose the heart to potentially fatal arrhythmias. Further investigations of class-selective HDACIs are necessary to understand the underlying mechanisms for arrhythmogenic adverse cardiac effects and improved cardiac safety profiles for this emerging class of novel therapeutics with diverse beneficial clinical indications.

## Conflict of Interest Statement

The authors declare that the research was conducted in the absence of any commercial or financial relationships that could be construed as a potential conflict of interest.
